# Oligopeptide Targeting Sortase A as Potential Anti-infective Therapy for *Staphylococcus aureus*

**DOI:** 10.3389/fmicb.2018.00245

**Published:** 2018-02-14

**Authors:** Jianfeng Wang, Hongen Li, Juan Pan, Jing Dong, Xuan Zhou, Xiaodi Niu, Xuming Deng

**Affiliations:** ^1^Center of Infection and Immunity, The First Hospital, Jilin University, Changchun, China; ^2^Key Laboratory of Zoonosis, Ministry of Education, Institute of Zoonosis, College of Veterinary Medicine, Jilin University, Changchun, China; ^3^Tianjin International Travel Healthcare Center, Tianjin, China

**Keywords:** *Staphylococcus aureus*, Sortase A, oligopeptide, antivirulence, mastitis

## Abstract

Sortase A (SrtA)-catalyzed anchorage of surface proteins in most Gram-positive bacteria is indispensable for their virulence, suggesting that this transpeptidase is a promising target for antivirulence therapy. Here, an oligopeptide, LPRDA, was identified as an effective inhibitor of SrtA via virtual screening based on the LPXTG substrate sequence, and it was found to inhibit SrtA activity *in vitro* and *in vivo* (IC_50_ = 10.61 μM) by competitively occupying the active site of SrtA. Further, the oligopeptide treatment had no anti-*Staphylococcus aureus* activity, but it provided protection against *S. aureus*-induced mastitis in a mouse model. These findings indicate that the oligopeptide could be used as an effective anti-infective agent for the treatment of infection caused by *S. aureus* or other Gram-positive bacteria via the targeting of SrtA.

## Introduction

Mastitis, or inflammation of the mammary gland, is a common and devastating disease that causes serious economic losses for the dairy industry worldwide (Duarte et al., 2015). The contamination of dairy products with antibiotic residues due to antibiotic therapy seriously affects milk quality and poses public health risks (Addo et al., 2011). Furthermore, antimicrobial therapy for this disease has been increasingly challenging due to drug resistance (Virdis et al., 2010). Among the etiological agents, *Staphylococcus aureus* is the most important and prevalent contagious mammary pathogen, and it causes other clinical infections ranging in severity from mild to fatal, including dermonecrotic skin infection, pneumonia, sepsis, and endocarditis (de Almeida et al., 2013; Zhang et al., 2015). The remarkable ability of *S. aureus* to transmit and acquire resistance to currently available antibiotics further amplifies the gravity of infections, including mastitis, caused by this pathogen, especially multi-drug-resistant strains, such as methicillin-resistant *S. aureus* (MRSA) (Tam et al., 2007; Ciustea et al., 2012). Thus, novel antibacterial agents for the treatment of infections, including mastitis, caused by antibiotic-resistant *S. aureus* are urgently needed.

To successfully establish infection, *S. aureus* produces a vast array of virulence factors, including surface proteins and secreted toxins and enzymes that facilitate bacterial adherence, tissue invasion and destruction, and host defense evasion (Foster et al., 2014; Jusko et al., 2014). Among these processes, bacterial adhesion and invasion mediated by surface proteins (fibronectin-binding proteins, adhesin, protein A, clumping factors, and collagen-binding proteins) are the initial steps that are essential for *S. aureus* infection (Foster et al., 2014). Additionally, surface proteins play important roles in the evasion of host immune attack (Maresso and Schneewind, 2008). Similar to other Gram-positive pathogens, the anchorage of many virulence-associated surface proteins to the cell wall of *S. aureus* is catalyzed by the transpeptidase Sortase A (SrtA, encoded by the *srtA* gene) via a mechanism requiring a C-terminal sorting signal with an LPXTG motif (Maresso and Schneewind, 2008; Bradshaw et al., 2015). SrtA recognizes and cleaves the LPXTG recognition motif between the threonine (T) and glycine (G) residues of the surface proteins and subsequently catalyzes amide bond formation between the resulting surface protein fragment and the Gly_5_ cross-bridges of the cell wall peptidoglycan (Jonsson et al., 2002; Bradshaw et al., 2015). Previous studies have described a *S. aureus* SrtA mutant that fails to process and display surface proteins and exhibits abnormal pathogenesis of animal infections (Mazmanian et al., 2000, 2002). Furthermore, the structure of SrtA is highly conserved among Gram-positive pathogens (Suree, 2009). Therefore, SrtA inhibitors may represent new agents with novel strategies that may be used in the battle against bacterial infections.

Several studies have reported that SrtA inhibitors, including natural and synthetic compounds, significantly attenuate *S. aureus* virulence both *in vitro* and *in vivo* (Suree, 2009; Zhang et al., 2014). Although a phosphinic peptidomimetic inhibitor has been demonstrated to inhibit *S. aureus* SrtA activity (McCafferty et al., 2004), the therapeutic effect of an oligopeptide against *S. aureus* infection *in vivo* has not yet been reported. Here, we designed and evaluated several oligopeptide inhibitors of SrtA based on the LPXTG substrate sequence by virtual screening. The oligopeptide LPRDA was chosen for further study due to its relatively high inhibitory activity. The potential mechanism underlying this inhibition was characterized by molecular dynamics (MD) simulations, and the therapeutic effect of the oligopeptide inhibitor against *S. aureus* virulence was further determined in an animal model of mastitis. This study is the first, to our knowledge, to identify an oligopeptide inhibitor of SrtA with effects against *S. aureus* mastitis *in vivo* that does not affect cell viability.

## Materials and Methods

### Bacterial Strains, Growth Conditions and Reagents

The MRSA strain USA 300 was obtained from the American Type Culture Collection (ATCC, Manassas, VA, United States) and grown at 37°C in tryptic soy broth (TSB). USA 300ΔsrtA was cultured in TSB supplemented with spectinomycin. The modified oligopeptide used in this study was synthesized by Scilight Biotechnology (Beijing, China) and was dissolved in phosphate-buffered solution (PBS). To improve its pharmacokinetics and therapeutic efficacy, the oligopeptide was modified by PEG2000 modification and amide modification at the N- and C-termini, respectively.

### Virtual Screening for SrtA Inhibitors

Virtual screening performance was carried out using Autodock vina software. According to previous literature (Zong et al., 2004; Suree et al., 2009), residues of C184, W194, and P197 in SrtA play the key role in the binding of substrate LPXTG with SrtA. Therefore, these residues are used as the binding sites of molecular docking. A grid box of dimensions (15 × 20 × 15) with a spacing of 1 Å was created and centered on the mass center of the ligand. The inhibitors were ranked according to the lowest energy representative from each binding sites.

Molecular modeling was used in this study for the virtual screening of potential inhibitors of SrtA (Gao et al., 2016). First, the initial structure of SrtA was obtained from the 3D X-ray structure (PDB code: 1T2P). To determine the starting structure of the ligand/SrtA complex for MD simulations, a standard docking procedure for a rigid protein and a flexible ligand was performed with AutoDock 4 (Hu et al., 2010). Subsequently, MD simulations of the complex system were performed; details of the computational biology method used have been previously described (Lv et al., 2013; Niu et al., 2013).

### Recombinant SrtA Protein Expression and Purification

An *Escherichia coli* BL21 (DE3) strain harboring a recombinant vector encoding SrtA_∆N59_ was used, as described previously (Wang et al., 2015c). Cells were cultured overnight at 16°C with 1 mM isopropyl-β-D-thiogalactopyranoside (IPTG, Invitrogen, Carlsbad, CA, United States) in TSB containing ampicillin to induce SrtA expression until the OD_600_ reached 0.6 ∼ 0.8. Then, the cells were harvested by centrifugation at 5,500 × *g* for 10 min, resuspended in reaction buffer (50 mM Tris-HCl, 5 mM CaCl_2_, and 150 mM NaCl, pH 7.5) and lysed by sonication. After centrifugation (16,000 × *g*, 30 min, 4°C), the supernatant was loaded onto a self-packaged GST-affinity column and was then washed with PBS. The bound GST-tagged proteins were incubated with Precision Protease at 4°C overnight prior to elution with reaction buffer. Purified SrtA proteins were concentrated to 10 mM via centrifugal filtration (Millipore, Bedford, MA, United States) and subsequently analyzed by SDS-PAGE.

The procedures followed for expression and purification of the mutant proteins (SrtA_N114A_, SrtA_C184A_, and SrtA_R197A_) were identical to those followed for the wild-type protein (SrtA_ΔN59_).

### Site-Directed Mutagenesis

Site-directed mutagenesis for SrtA_N114A_, SrtA_C184A_, and SrtA_R197A_ was conducted using a recombinant vector encoding SrtA_ΔN59_ with a QuickChange Site-Directed Mutagenesis Kit (Stratagene, La Jolla, CA, United States). The primer pairs for these three mutations were as follows: N114A forward, 5′-GAATCACTAGATGATCAAGCGATTTCAATTGCAGGACAC-3′; N114A reverse, 5′-GTGTCCTGCAATTGAAATCGCTTGATCATCTAGTGATTC-3′; C184A forward, 5′-CAATTAACATTAATTACTGCGGATGATTACAATGAAAAG-3′; C184A reverse, 5′-CTTTTCATTGTAATCATCCGCAGTAATTAATGTTAATTG-3′; R197A forward, 5′-GGCGTTTGGGAAAAAGCGAAAATCTTTGTAGC-3′; and R197A reverse, 5′-GCTACAAAGATTTTCGCTTTTTCCCAAACGCC-3′.

### SrtA Activity Inhibition Assay

The inhibitory effect of the oligopeptide against SrtA and its mutants activity was determined as previously described in 96-well black plates (PerkinElmer, Boston, MA, United States) (Wang et al., 2015c). Briefly, 100 μL of purified SrtA (4 mM) was preincubated with 100 μL of increasing concentrations of the oligopeptide (3.125–25 μM) in reaction buffer at 37°C for 20 min. The model fluorescent peptide substrate Dabcyl-QALPTTGEE-Edans (GL Biochem, Shanghai, China) (final concentration of 10 μM) was added to the reaction system, followed by incubation at 37°C for 1 h. Fluorescence intensity was measured at emission and excitation wavelengths of 350 and 520 nm, respectively. The experiments were repeated three times independently for each sample.

### Anti-*S. aureus* Activity of Oligopeptide

The broth microdilution method was used to determine the minimum inhibitory concentration (MIC) of the oligopeptide against the MRSA strain USA 300 according to the NCCLS guideline M31-A2. For the growth curve experiment, an overnight bacterial culture was inoculated in fresh TSB (1:100) with the indicated concentrations of the oligopeptide, and the optical density (OD) at 600 nm was measured for each sample at 1-h intervals.

### Construction of *srtA* Deletion Mutant of USA 300

The *srtA* gene of *S. aureus* USA 300 was inactivated by allelic exchange as previously described (Chen et al., 2014). Briefly, two DNA fragments were PCR amplified from the USA 300 genome using the primers Down-srtA-f (GCGGAATTCTCTTTTCATCTTTATCTTTATCGTG) and Down-srtA-r (GCGGGATCCAAATACAAAGATAGGCTCAT), as well as Up-srtA-f (GCGGTCGACCCGTAGAAAAAATATTGAATAA) and Up-srtA-r (GC GCCATGGTAAGAGATGTTAAGCCTACA). A 1.5-kb fragment including the spectinomycin resistance gene was PCR amplified using the primers Spc-f (CATGCCATGGGTTCGTGAATACATGTTATA) and Spc-r (CCGGAATTCGTTTTCTAAAATCTGAT) from a pSET2 plasmid. These three fragments were then mixed, digested with EcoRI and NcoI, and ligated at 4°C for 1 h. Using the primers Up-srtA-f and Down-srtA-r, a 2.4-kb fragment of the ligated product was PCR amplified, digested with BamHI and SalI, inserted into pBT2, and used for allele replacement as previously described. The presence of the mutation was confirmed by PCR sequencing analysis based on the known sequences of USA 300 and its SrtA mutants. The strA-knockout strain showed normal growth in TSB.

### Immunofluorescence Assay

Detection of surface protein A by immunofluorescence analysis was performed as described previously (Chen et al., 2014). Briefly, *S. aureus* were cultured in TSB with or without the oligopeptide at 37°C for 2.5 h, pelleted by centrifugation (3,000 × *g*, 10 min), washed three times with PBS, blocked with 2% BSA for 1 h at 37°C, stained with fluorescein isothiocyanate (FITC)-labeled goat anti-rabbit IgG for 2 h at 37°C, and observed under a laser scanning confocal microscope (Olympus, Tokyo, Japan).

### Fibronectin-Binding Assay

Ninety-six-well flat-bottom polystyrene plates were coated with 100 μL of 2 μg/mL fibronectin in PBS (fibronectin from bovine plasma; Sigma–Aldrich, St. Louis, MO, United States) per well and incubated at 4°C overnight. Each well was washed three times with PBS and blocked with 5% BSA in PBS at 37°C for 2 h. An overnight bacterial culture was inoculated in fresh TSB (1:100, OD_600 nm_ ≈ 0.2) with the indicated concentrations of the oligopeptide and incubated until the OD_600 nm_ reached 0.5. Then, the cells were centrifuged at 5000 × *g* for 10 min and resuspended in PBS to an OD_600 nm_ of 1.0. One hundred microliters of bacterial suspension were added to each fibronectin-coated plate well as described above, and the plates were incubated at 37°C for 2 h. After removal of the cell suspension, 25% (v/v) formaldehyde was added to fix *S. aureus*. Then, the bacteria attached to fibronectin were stained with 100 μL of 0.4% crystal violet dye for 20 min at 37°C after being washed once with PBS. Following removal of the supernatant, adherence was quantified by measuring the OD_570 nm_ for each well. USA 300 without the oligopeptide was used as a positive control, and PBS was used as a negative control.

### Biofilm Formation Assay

An overnight bacterial culture was standardized to an OD_600 nm_ of 0.01 in TSB supplemented with the indicated concentrations of the oligopeptide. One hundred microliters of *S. aureus* (USA 300 and its SrtA mutants) suspension were added to the wells of microtiter plates and incubated for 24 h at 37°C. Then, the suspension was removed, and the plates were washed twice with PBS. Adherent biofilms were fixed with 100% ethanol, stained with 0.1% (v/v) crystal violet, washed several times with PBS, and quantitatively assessed by adding 100% ethanol and measuring the OD_570 nm_ for each well. USA 300 without the oligopeptide was used as a positive control, and PBS was used as a negative control.

### Invasion Assay

J774 cells were adjusted to 4 × 10^5^ per glass slide in 24-well plates in Dulbecco’s minimum essential medium (DMEM) supplemented with 10% fetal bovine serum (FBS) and cultured in an incubator at 5% CO_2_ and 37°C overnight. An overnight bacterial culture was inoculated in fresh TSB (1:100, OD_600 nm_ ≈ 0.2) with the indicated concentrations of the oligopeptide and incubated until the OD_600 nm_ reached 1.0. Then, the bacteria were resuspended in DMEM containing 10% FBS at a concentration of 1 × 10^8^ CFU/mL, and 1 mL of the resuspension was added to each well for infection at 37°C for 1 h. Invasion was terminated by incubation with 300 μg/mL gentamicin for 30 min. After three washes with PBS, coverslips were placed in 0.2% Triton X-100 with vigorous vortexing, and the diluted lysates were plated onto TSB agar plates to enumerate the number of bacteria associated with each sample.

### Animal Experiments

BALB/c mice (from the Experimental Animal Center of Jilin University) were obtained and housed under specific pathogen-free conditions. All animal studies were approved by and conducted in accordance with the guidelines of the Animal Welfare and Research Ethics Committee of Jilin University. An overnight bacterial culture was inoculated in fresh TSB broth (1:100, OD_600 nm_ ≈ 0.2) and incubated until the OD_600 nm_ reached 0.8. Cells were harvested by centrifugation at 3,000 × *g* for 10 min, washed three times with PBS, and resuspended in PBS to a concentration of 1 × 10^9^ CFU/mL for the mastitis infection experiment.

For establishment of a mastitis model of infection, lactating BALB/c mice, 10–12 weeks of age and weighing 30–32 g, were anesthetized using sodium pentobarbital and infected by injecting the canal glands with 50 μL of staphylococcal suspension per breast. The infected mice were administered 50 mg/kg of the oligopeptide subcutaneously at the time of infection and then at 12-h intervals. The control mice received an equal volume of PBS at the same time points. To evaluate the pathological correlates of mastitis, mammary gland tissues isolated from sacrificed mice at 48 h post-infection were fixed in 4% paraformaldehyde, stained with hematoxylin and eosin, and visualized by light microscopy. Furthermore, additional mammary gland tissues from the mice sacrificed at 48 h post-infection were homogenized in PBS containing 2% Triton X-100, after which they were appropriately diluted and inoculated onto TSB agar plates for assessment of the bacterial burden. The levels of the inflammatory cytokines IL-1β, IL-6, and TNF-α in the supernatants were measured using ELISA kits.

### Determination of Binding Affinities of Ligands with Proteins

In this study, the fluorescence quenching method was used to measure the binding constants (*K*_A_) of ligands to proteins. A 280-nm excitation wavelength with 5-nm band pass and a 345-nm emission wavelength with 10-nm band pass were used for the measurements. Details of the measurement procedures have been described previously (Bandyopadhyay et al., 2002; Jurasekova et al., 2009).

### Statistics

The data are presented as the mean ± SD (*n* ≥ 3). Student’s *t*-test for paired sample was used to determine statistical significance using SPSS 13.0. *P* ≤ 0.05 was considered to be statistically significant.

## Results

### Deletion of *srtA* in USA 300

**FIGURE 1:**
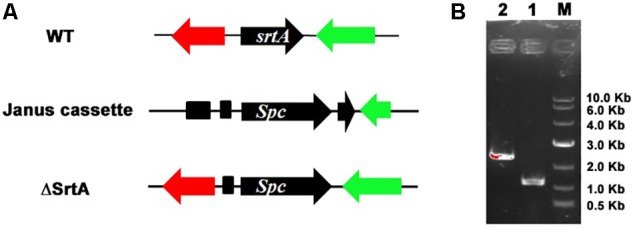
Mutation of *srtA* in *Staphylococcus aureus* USA 300. **(A)** The intact *srtA* gene in *S. aureus* USA 300 was disrupted by recombination deletion with a Janus cassette. **(B)** The *srtA* mutation was confirmed by PCR based on the chromosomal DNA sequences in *S. aureus* USA 300 and its SrtA mutant strain ΔSrtA. PCR products for the parent strain USA 300 (lane 1) and its mutant strain ΔSrtA (lane 2).

To further analyze the role of SrtA in *S. aureus* virulence, we first inactivated the *srtA* gene in the *S. aureus* USA 300 strain by homologous recombination (**Figure 1A**), as confirmed by PCR sequencing analysis of chromosomal DNA from the *S. aureus* USA 300 strain and its SrtA mutants (**Figure 1B**).

### Screening and Validation of SrtA Inhibitors

**FIGURE 2:**
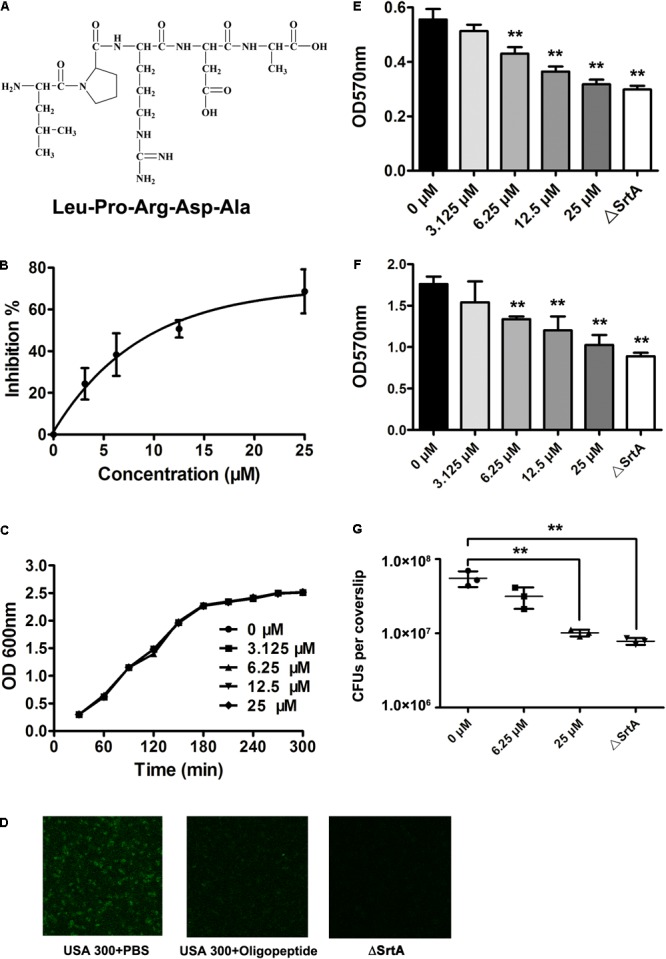
Inhibition of SrtA activity both *in vitro* and *in vivo* by the oligopeptide. **(A)** The molecular structure of the oligopeptide, LPRDA. **(B)** The inhibitory effect of the oligopeptide on SrtA activity. The fluorescent peptide substrate Dabcyl-QALPTTGEE-Edans was mixed with purified SrtA preincubated with various concentrations of the oligopeptide, and the fluorescence intensity of the reaction system was determined at emission and excitation wavelengths of 350 and 520 nm, respectively (*n* = 3). **(C)** The growth of *S. aureus* USA 300 cultured in TSB in the presence of increasing concentrations of the oligopeptide was monitored by measuring the OD at 600 nm every 60 min. Oligopeptide treatment inhibits the binding of *S. aureus* to fibronectin **(D)** and the formation of *S. aureus* biofilm **(E)** (*n* = 3) (^∗^*p* < 0.05, ^∗∗^*p* < 0.01). **(F)** Inhibition of *S. aureus* invasion into J774 cells by the oligopeptide. J774 cells were infected with *S. aureus* at 37°C for 1 h, and the total colony-forming units (CFU) for lysed cells was evaluated (*n* = 3) (^∗^*p* < 0.05, ^∗∗^*p* < 0.01). **(G)** The presence of protein A on the cell envelopes of *S. aureus* treated with or without the oligopeptide and its SrtA mutant was viewed under a confocal laser scanning microscope following staining with an FITC-conjugated antibody. Results are reported as mean ± SEM.

The docking of a series of ligands, based on the LPXTG substrate sequence, into the SrtA active site was carried out, and their binding affinities were estimated using the Autodock vina package for virtual screening of the SrtA inhibitors. As shown in **Table 1**, the binding affinity between the oligopeptide LPRDA (**Figure 2A**) and SrtA was higher than those of all other oligopeptides tested, indicating that this oligopeptide may have the strongest inhibitory activity against SrtA. Thus, this oligopeptide was chosen for further validation of the inhibitory activity in the following experiments. A fluorescence assay was employed to assess the putative inhibition of SrtA activity by the oligopeptide based on the model peptide substrate Dabcyl-QALPTTGEE-Edans. Consistent with the computational screening results, the catalytic activity of the reaction mixture exposed to the oligopeptide was significantly decreased (**Figure 2B**). Importantly, the concentration required for 50% inhibition (IC_50_) was 10.61 μM under our experimental conditions (**Figure 2B**), indicating that this oligopeptide is a relatively highly efficient inhibitor of *S. aureus* SrtA compared with the inhibitors tested in previous studies (Cegelski et al., 2002; Zhang et al., 2014). Additionally, the MIC of the tested oligopeptide for MRSA USA 300 was greater than 200 μM, and the growth of *S. aureus* was not visibly affected in the presence of the oligopeptide at concentrations required to inhibit SrtA catalytic activity (3.125–25 μM) (**Figure 2C**). Taken together, these results indicate that the oligopeptide LPRDA is an effective SrtA inhibitor without anti-*S. aureus* activity, suggesting that it may be a promising antivirulence candidate for anti-infective therapy.

**Table 1:** The screening and validation of SrtA peptide inhibitors.

Oligopeptide	Binding energy
LPRDA	–6.9 kcal/mol
LPTTG	–5.6 kcal/mol
LPESG	–4.9 kcal/mol
LTESG	–5.5 kcal/mol
ISESG	–6.1 kcal/mol
IPESG	–5.8 kcal/mol
ISELG	–6.2 kcal/mol
ISEVG	–6.0 kcal/mol
ISEQG	–5.7 kcal/mol
LPIDA	–5.5 kcal/mol

### Oligopeptide Inhibits SrtA Activity in *S. aureus*

Sortase A has been shown to be a key enzyme that catalyzes the anchoring of surface proteins in most Gram-positive bacteria (Cascioferro et al., 2014). Thus, we hypothesized that treatment with the oligopeptide might inhibit the display of surface proteins on *S. aureus* and subsequently trigger reduced capacities for adhesion, biofilm formation and invasion of this pathogen. Consistent with previous results, the display of surface protein A was only confirmed for wild-type *S. aureus*, as demonstrated by its tinctorial strength, and the mutant strain was defective in displaying this surface protein (**Figure 2D**). However, addition of the oligopeptide significantly hindered the binding of FITC-conjugated IgG to SrtA, indicating effective inhibition of the anchoring of surface protein A by the oligopeptide treatment (**Figure 2D**). Adhesion and biofilm formation of *S. aureus*, as mediated by surface proteins, are important for prevention to be eradicated by the host. Thus, fibronectin-binding and biofilm formation assays were employed to assess the effects of the oligopeptide on these processes in *S. aureus.* In contrast with the weak adherence of the SrtA mutant, USA 300 exhibited strong adherence to bovine fibronectin (**Figure 2E**). Significantly, treatment of *S. aureus* with the oligopeptide resulted in decreased adherence to the fibronectin coating on the well bottom (**Figure 2E**). A similar phenomenon of inhibition was also observed in the biofilm formation assay (**Figure 2F**), in agreement with the findings of inhibition of SrtA activity and *S. aureus* fibronectin-binding activity by the oligopeptide. Furthermore, the invasion of *S. aureus* into J774 cells was examined to determine the potential inhibitory effect of the oligopeptide. As expected, the wild-type *S. aureus* strain exhibited a strong invasion ability compared with the SrtA mutant strain, and significant anti-invasive activity of *S. aureus* was observed following treatment with 25 μM oligopeptide (**Figure 2G**). Taken together, these results indicate that the oligopeptide treatment inhibited SrtA activity in *S. aureus* and subsequently hindered bacterial adhesion, biofilm formation, and invasion.

### Oligopeptide Inhibits *S. aureus* Virulence in Animal Model of Infection

**FIGURE 3:**
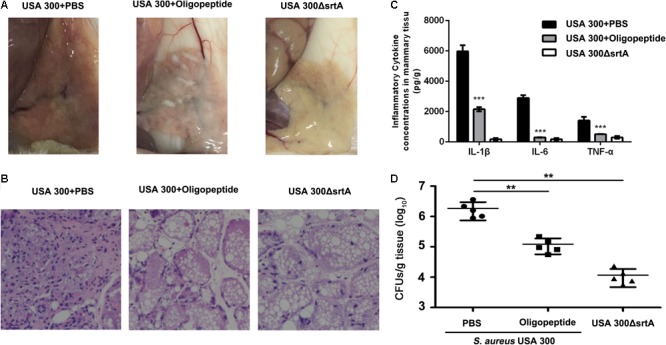
Oligopeptide provides protection against *S. aureus* mastitis in a mouse model. Lactating BALB/c mice were infected by injecting the canal glands with 5 × 10^7^ CFU bacterial cells and treatment with the oligopeptide or PBS at the time of infection. Gross pathological changes **(A)** and histopathological analysis **(B)** of the mammary gland tissues at 48 h post-infection (*n* = 10). Significant alleviation of the pathological abnormalities was observed in the infected mice that received 50 mg/kg oligopeptide compared with the control mice treated with PBS. **(C)** Oligopeptide reduces the inflammatory response in infected mice. The levels of cytokines, including IL-1β, IL-6, and TNF-α, in the mammary gland tissues of infected mice were evaluated by ELISA (*n* = 10) (^∗^*p* < 0.05, ^∗∗∗^*p* < 0.001). **(D)** The effect of the oligopeptide on the bacterial burden in infected mice. Mammary gland tissues were collected, homogenized, and plated on TSB agar plates for assessment of the bacterial burden (*n* = 5) (^∗^*p* < 0.05, ^∗∗^*p* < 0.01). Results are reported as mean ± SEM.

The inhibition of SrtA activity both *in vitro* and in *S. aureus* by the oligopeptide, in addition to the critical role of SrtA in *S. aureus* virulence, led us to reason that the SrtA inhibitor oligopeptide would also be an effective candidate for suppression of *S. aureus* infection. Here, the therapeutic effect of the oligopeptide against mice mastitis was evaluated. Following challenge with *S. aureus*, the breasts of the lactating mice were injected with the oligopeptide or PBS. To evaluate the impact of the oligopeptide treatment, histopathologic analysis of mammary gland tissues at 48 h post-infection was performed. Consistent with a previous study, almost no inflammatory responses or pathological changes were detected in the mice infected with the *S. aureus* SrtA mutant (**Figures 3A,B**), while significant inflammatory responses were observed in the mice infected with *S. aureus* (**Figures 3A,B**), with destroyed or abnormal mammary glands, dense inflammatory cells, and depletion of epithelial cells in the mammary gland tissues (**Figures 3A,B**). However, the oligopeptide treatment effectively alleviated the *S. aureus*-induced inflammatory responses and pathological changes in the infected mice, which exhibited only mild tissue injury (**Figures 3A,B**), demonstrating a potential prophylactic effect of the oligopeptide against *S. aureus*-induced mastitis. Further, the bacterial counts in the mammary gland tissues indicated a significant decrease in the bacterial burden in the oligopeptide-treated mice compared with the PBS-treated control mice (**Figure 3D**). Consistent with these observations, the levels of cytokines, including IL-1β, IL-6, and TNF-α, in the *S. aureus*-infected mice were markedly increased compared with those in the mice infected with the *S. aureus* SrtA mutant (**Figure 3C**), whereas administration of the oligopeptide significantly reduced the *S. aureus*-mediated increases in the cytokine levels (**Figure 3C**). Taken together, these results indicated that treatment with the oligopeptide LPRDA conferred effective protection against *S. aureus*-induced mastitis in the mouse model.

### Determination of the Molecular Mechanism of Oligopeptide Effects against SrtA

**FIGURE 4:**
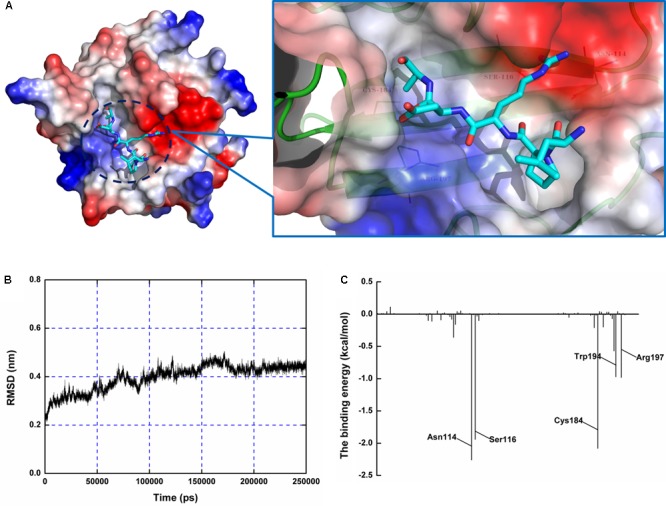
Determination of the 3D structure of the SrtA-oligopeptide complex using a molecular modeling method. **(A)** The structure of SrtA-oligopeptide. **(B)** The RMSD calculated for the backbone atoms of the protein during MD simulations of the SrtA-oligopeptide is presented. **(C)** Decomposition of the binding energy on a per-residue basis at the binding sites of the SrtA-oligopeptide complex.

**FIGURE 5:**
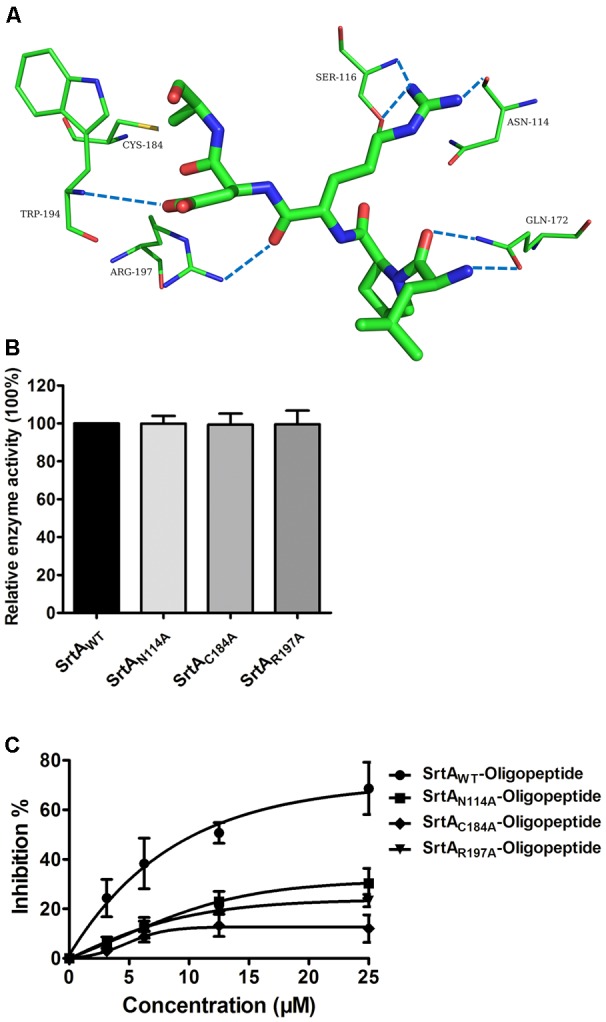
Determination of the binding sites for the oligopeptide on SrtA and its inhibitory mechanism. **(A)** The predicted binding mode of SrtA with the oligopeptide is shown, with labeling of key residues in the binding sites. **(B)** The catalytic activity of SrtA and its derivative. The activity of SrtA mutants was calculated by comparison with the wild type SrtA activity that was set to 100%. **(C)** Influences of the oligopeptide on the activities of SrtA and its derivatives. SrtA was incubated with various concentrations of the oligopeptide, and the fluorescent peptide substrate Dabcyl-QALPTTGEE-Edans was added for determination of SrtA protein activity, as described in **Figure 1B** (*n* = 3). Results are reported as mean ± SEM.

Using a computational biology method, the potential binding mode of the oligopeptide with SrtA at the active site was explored in this study. As is shown in **Figure 4**, the complex was found to reach equilibrium at 100 ns based on analysis of the root-mean-square deviations (RMSDs) of the backbone C_α_ atoms. The binding of the oligopeptide to SrtA and the binding mode are presented in **Figure 5A**. It was clear that the oligopeptide bound to SrtA via hydrogen bonding and hydrophobic interactions. During the time course of the simulation, the oligopeptide localized to the catalytic pocket of SrtA (residues 90–120 and 180–200). In detail, the binding model of the oligopeptide with SrtA revealed that the Arg, Leu, and Asp side chains of the oligopeptide formed seven hydrogen bonds with Asn114, Ser116, Gln172, Trp194, and Arg197, respectively. The relevant information concerning the stability of the hydrogen bonds between SrtA and oligopeptide are summarized in **Table 2**.

**Table 2:** SrtA-oligopeptide H-bonds from MD simulations.

Acceptor	Donor	Presence %	Distance (Å)
Oligo: Arg N	SrtA: Asn114 O-H	77.8	2.3
Oligo: Arg N	SrtA: Ser116 O-H	71.4	1.9
Oligo: Arg N	SrtA: Ser116 N-H	69.8	1.8
Oligo: Leu O	SrtA: Gln172 N-H	77.4	2.8
SrtA: Gln172 O	Oligo: Leu N-H	66.4	3.6
Oligo: Asp O	SrtA: Trp194 N-H	68.1	3.3
Oligo: Arg O	SrtA: Arg197 N-H	69.5	2.8

To examine the energy contributions from the residues of the binding sites in the SrtA-oligopeptide complex, energy decomposition was analyzed for the SrtA-oligopeptide complex system. As shown in **Figure 4C**, Asn114, Ser116 and Cys184 had a strong total binding energy contribution, with Δ*E*_total_ of ≤-2.0 kcal/mol. The residues Trp194 and Arg197 also had an appreciable total binding energy contribution, with Δ*E*_total_ of ≤-1.0 kcal/mol. These results suggest that these five residues are key residues in the sortaseA.

To confirm these results, the total binding free energy for the SrtA-oligopeptide complex and the detailed energy contributions were calculated according to the MM-PBSA approach, as summarized in **Table 3**. According to the results, the binding free energy, Δ*G_bind_*, of the interaction between the oligopeptide and protein decreased in the order of SrtA_WT_ > SrtA_Mutants_, indicating that SrtA_WT_ exhibited the strongest binding to the oligopeptide. Further, we measured the Δ*G_bind_* and the number of binding sites between the oligopeptide and the three mutants using the fluorescence spectroscopy quenching method, and the results were highly consistent with those obtained using computational methods (**Table 3**). These results indicated that the information generated by the MD simulations on the SrtA-oligopeptide complex was reliable. To further confirm the molecular mechanism, we assessed the inhibitory effects of the oligopeptide against SrtA and its mutants. All the three mutants displayed the similar catalytic activity as wild-type SrtA (**Figure 5B**); however, the sensitivity of the SrtA mutants to oligopeptide-induced inhibition was significantly decreased compared to that of wild-type SrtA (**Figure 5C**), consistent with the fluorescence spectroscopy quenching assay results. Thus, these results demonstrated that binding of the oligopeptide inhibitor to the active region (residues Asn114, Ser116, Gln172, Cys184, Trp194, and Arg197) resulted in inhibition of the biology activity of SrtA.

**Table 3:** The binding free energy (kcal/mol) of SrtA_WT_-oligopeptide, SrtA_N114AT_-oligopeptide, SrtA_C184A_-oligopeptide, and SrtA_R197A_-oligopeptide systems based on a computational method and the binding constant (*K*_A_) values based on fluorescence spectroscopy quenching.

	SrtA_WT_	SrtA_N114A_	SrtA_C184A_	SrtA_R197A_
Computational method	–11.4 ± 2.1	–7.1 ± 1.4	–7.7 ± 1.1	–7.9 ± 1.6
*K*_A_ (1 × 10^4^) L⋅mol^-1^	6.6 ± 1.3	4.2 ± 1.1	5.1 ± 1.2	5.0 ± 1.4

## Discussion

The development of drug resistance (Rasko and Sperandio, 2010) promote the alternative antivirulence strategy for the treatment of bacterial infections, especially those caused by resistant strains (Zambelloni et al., 2015). Recent research efforts have been directed at discovery of small molecules and antibodies that inhibit the expression or neutralize the functions of virulence-associated toxins (Bubeck Wardenburg and Schneewind, 2008; Wang et al., 2015a,b). However, SrtA-anchored surface proteins in the bacterial envelope are indispensable for bacterial adhesion, invasion and evasion in the host, and these processes are the first steps as well as the prerequisites for success in establishing infection (Cossart and Jonquieres, 2000; Maresso and Schneewind, 2008; Foster et al., 2014). Thus, the targeting of SrtA is more effective than that of the virulence-associated toxins (Cascioferro et al., 2014).

In the present study, virtual screening based on the LPXTG substrate sequence was used to screen for *S. aureus* SrtA inhibitors, among which the oligopeptide LPRDA exhibited the highest inhibitory effect against SrtA activity, with an IC_50_ of 10.61 μM. Importantly, the display of surface protein A by USA 300 was decreased in the presence of the oligopeptide. Subsequently, the interaction of *S. aureus* with fibronectin, the biofilm formation of *S. aureus*, and the invasion of *S. aureus* into host cells were all inhibited in our experimental conditions following treatment with the oligopeptide, indicating that suppression of SrtA activity by the oligopeptide in *S. aureus* also occurred. Furthermore, the oligopeptide had no bacteriostatic activity against *S. aureus* at the concentrations at which it sufficiently inhibited SrtA activity both *in vitro* and *in vivo*, suggesting that this inhibitor should be able to inhibit *S. aureus* virulence without exerting noticeable selective pressure. Treatment with the oligopeptide remarkably alleviated *S. aureus*-mediated pathological injury and inflammatory reactions in the mouse model of mastitis infection; this disease is considered the most frequent and most costly disease affecting the dairy industry. Clinical treatment failure frequently occurs with the use of antibiotics due to the development of drug resistance, and the presence of antibiotic residues in milk significantly reduces its quality and has undesirable effects on human health (Testa et al., 2007; Virdis et al., 2010; Duarte et al., 2015). Although some natural and synthetic compounds targeting virulence factors, including SrtA, have been identified as therapeutic agents for this disease (Cascioferro et al., 2014; Zhang et al., 2014; Wang et al., 2015c), the excretion of these compounds into milk also affects its quality (Addo et al., 2011). In the present study, treatment of mastitis with the oligopeptide may result in decreased drug resistance compared with that caused by the currently available drugs because of its low antibacterial activity (Zambelloni et al., 2015). Moreover, the breakdown products of the oligopeptide in the host are amino acids, which are the basic components of proteins, ensuring for production of high-quality milk, as no antibiotic residues are deposited. Therefore, the oligopeptide LPRDA may be a candidate for use in an alternative strategy for the prevention and treatment of mastitis.

Additionally, MD simulations were carried out for the SrtA-oligopeptide complex system to explore the interaction mechanism between the oligopeptide LPRDA and SrtA. This simulation revealed that the oligopeptide localized to the catalytic pocket of SrtA (residues 90–120 and 180–200), very close to the substrate binding site. Due to binding of the oligopeptide with SrtA, binding of the substrate with SrtA was blocked, leading to the loss of biological activity of SrtA. Fluorescence spectroscopy quenching assay and SrtA inhibition assay were performed to validate this inhibitory mechanism. As expected, the binding free energies of the SrtA mutants with the oligopeptide were significantly lower than that of wild-type SrtA, and the inhibitory effects of the oligopeptide against the SrtA mutants were decreased compared with that of wild-type SrtA. Our data established that inhibition of SrtA activity by the oligopeptide LPDRA via occupation of the active site of this enzyme conferred protection against *S. aureus* mastitis. Considering the indispensable role of SrtA in Gram-positive bacterial pathogenicity and the conserved structure of its active site (Jonsson et al., 2002; Mazmanian et al., 2000, 2002; Chen et al., 2014), the oligopeptide LPDRA should prevent a variety of bacterial infections requiring SrtA-catalyzed surface protein anchoring, especially those caused by resistant Gram-positive bacteria.

## Author Contributions

XD, XN, JW, and HL conceived and designed the experiments. JW, HL, JP, JD, and XZ performed the experiments. JW and XD contributed reagents/materials/analysis tools. JW and XD wrote the paper.

## Conflict of Interest Statement

The authors declare that the research was conducted in the absence of any commercial or financial relationships that could be construed as a potential conflict of interest.
